# Reward anticipation selectively boosts encoding of gist for visual objects

**DOI:** 10.1038/s41598-020-77369-4

**Published:** 2020-11-19

**Authors:** Liyana T. Swirsky, Ryan M. Marinacci, Julia Spaniol

**Affiliations:** Department of Psychology, Ryerson University, 350 Victoria Street, Toronto, ON M5B 2K3 Canada

**Keywords:** Human behaviour, Motivation

## Abstract

Reward anticipation at encoding enhances later recognition, but it is unknown to what extent different levels of processing at encoding (gist vs. detail) can benefit from reward-related memory enhancement. In the current study, participants (*N* = 50) performed an incidental encoding task in which they made gist-related or detail-related judgments about pairs of visual objects while in anticipation of high or low reward. Results of a subsequent old/new recognition test revealed a reward-related memory benefit that was specific to objects from pairs encoded in the attention-to-gist condition. These findings are consistent with the theory of long-axis specialization along the human hippocampus, which localizes gist-based memory processes to the anterior hippocampus, a region highly interconnected with the dopaminergic reward network.

## Introduction

Our visual long-term memories result from encoding at multiple levels of analysis, ranging from visual detail to semantic gist^1^. If you pass a ladder on your walk to work you may process its particular color and orientation (detail-level analysis), or you may process it as a means of fixing the damaged roof upon which it leans (gist-level analysis). According to the levels of processing framework, gist-level analysis of semantic information at encoding is better than detail-level analysis of perceptual information for later recognition. Motivational states at encoding are likely to impact the fate of these memory representations. Past studies have shown that anticipation of reward during incidental encoding of visual objects is associated with enhanced subsequent recognition and recall^2,3^. Which level of memory processing drives such reward effects is less clear. Does reward motivation boost detail-level encoding representations, gist encoding representations, or both? The current study addressed this question using a behavioural-experimental approach. Its design and predictions were guided by current theories regarding the neuroanatomy of different levels of memory representation, and about neuromodulatory systems involved in motivation-cognition interactions.

### Levels of processing framework

The levels of processing framework^4–6^ describes the strength of a memory trace as a function of depth of processing. By this account, memory encoding takes place over a continuum ranging from sensory processing of low-level perceptual information to higher-order cognitive analysis of semantic information. Retention increases as a function of depth of processing such that information encoded at deeper, semantic levels will be better remembered than information encoded at shallower, perceptual levels^4^.

In intentional encoding paradigms, when participants are aware that their memory will be tested, various encoding strategies may be applied to commit information to memory. However, in incidental encoding tasks, the material is processed in a manner consistent with the task instructions^4^. When orienting instructions prompt participants to attend to the semantic gist of information rather than to perceptual details, memory outcomes tend to be more favourable. For example, sentence recall is higher when sentences are incidentally encoded with attention to their meaning rather than syntactic structure^7^ or constituent words^8^. Word recognition and recall is better for words that are incidentally learned while participants attend to their semantic rather than structural features^9–12^. Likewise, both recollection and familiarity are improved for words incidentally encoded with attention to meaning rather font^13^, and faces are better recognized when participants had to judge their potential occupation rather than their orientation^14^. In all of these examples, gist-level analysis at encoding provides better memory outcomes than detail-level analysis at encoding. This pattern is also consistent with fuzzy trace theory^15,16^, which predicts that gist-level memory traces are more durable than detail-level (i.e., verbatim) memory traces.

### Reward anticipation and memory

Another factor that can alter encoding processes to influence later memory outcomes is reward motivation. Reward anticipation, a motivational state induced by cues signaling the potential for reward, reliably elicits a dopaminergic response^17,18^ and can influence hippocampal-dependent memory via dopaminergic transmission from the midbrain and limbic regions to the hippocampus^19–21^. Indeed, reward anticipation during encoding boosts memory outcomes. For example, when tested at a 3-week delay, healthy adults had better recollection and source memory for pictures of objects that had predicted monetary reward, compared to neutral objects, during an incidental encoding task^21^. Similarly, when tested at a 24-h delay, healthy adults had better recognition of objects that were preceded by high-value reward cues, compared to low-value cues, during an intentional encoding task^22^. In another study, anticipation of high relative to low reward during incidental encoding was not only linked to improved recognition of target objects from the encoding task, but also improved memory for their associated background^2^. Each of these studies linked mesolimbic activation during encoding, or directly after, to successful delayed memory^2,21,22^.

Memory enhancement from reward anticipation has been demonstrated with many different paradigms. Behavioural and brain-based findings have linked reward anticipation to better memory for items encoded during anticipation of financial rewards^22^ and virtual incentives (e.g., value-directed remembering^23^) using paradigms for both incidental encoding^2,3,21^ and intentional encoding^22,24,25^ with tests of immediate recall^26^ and delayed recognition^25^. This suggests that reward anticipation from a variety of motivational incentives can enhance encoding and consolidation processes to boost memory.

A key aspect of reward anticipation is physiological arousal^27^. Arousal is associated with norepinephrine release in the locus coeruleus, which has widespread projections throughout the brain^28^. An influential recent framework, Glutamate Activates Noradrenergic Effects (GANE) theory^29^, proposes that the interaction of glutamatergic and noradrenergic activity during arousal increases the selectivity of perceptual, attentional, and encoding processes. As a result, arousal amplifies processing of salient stimuli while suppressing nonsalient stimuli^29,30^. These predictions have been supported in studies featuring a range of arousal manipulations and outcome measures. One study used an attention task that featured face stimuli made salient by a colored border, and presented alongside nonsalient house stimuli. Arousal, experimentally induced by tones that predicted the delivery of an electric shock, was associated with increased activation of face-sensitive brain regions, but decreased activation of house-sensitive brain regions^31,32^. Similarly, in a study focusing on mnemonic effects of arousal induced by emotional images, it was found that arousal boosted memory for neutral images when these images were task-relevant (i.e., salient). By contrast, arousal impaired memory for neutral images when these were task-irrelevant^33^.

In summary, like different levels of processing, reward anticipation can alter encoding processes by establishing states of motivation and arousal that guide attention. Both motivational incentives and arousing stimuli have been linked to improved memory outcomes. One putative mechanism common to both motivation and arousal is boosted selectivity of attention and memory, such that task-relevant target information is prioritized and task-irrelevant information is suppressed. It is not clear, however, how reward motivation at encoding would influence different levels of processing. On the one hand, motivation and arousal may act to boost encoding of both gist and detail when they are congruent with task instructions. On the other hand, reward may further bias attention and encoding to more meaningful, gist-level representations. A potential point of convergence for memory benefits of gist-level encoding and reward anticipation comes from the proposal of functional specialization along the longitudinal axis of the hippocampus^34^.

### Functional specialization for gist and reward within the hippocampus

The hippocampus plays a central role in episodic memory, and a recent proposal suggests a coarse-to-fine representational scale along the hippocampal long axis^34,35^. In this view, gist-based memory representations rely on anterior hippocampus^36–42^ whereas detailed representations rely on posterior hippocampus^43,44^. For example, rodent place cell receptive fields increase from smallest to largest along the dorsal–ventral hippocampal axis (akin to posterior-anterior in humans), suggesting that local and global representations are encoded at dorsal and ventral poles, respectively^45,46^. In humans, fMRI activation of anterior hippocampus activation during retrieval is associated with correct category-level recognition, indicating that anterior hippocampus supports gist memory^38^. Similarly, anterior, but not posterior, hippocampal activation has been shown to predict the verbatim effect—better gist vs. verbatim memory for sentences^41^—and volume loss in posterior hippocampus has been linked to greater reliance on gist-level memory representations over contextually-rich memories^39^. Finally, encoding of high-level, nested multi-event memories is associated with activity in anterior hippocampus, whereas encoding of more specific, single-event memories is associated with posterior hippocampus^37^. In summary, both animal studies and human neuroimaging studies provide converging evidence in support of a representational gradient along the longitudinal hippocampal axis.

Critically, there is also evidence for differential connectivity of anterior and posterior hippocampus with the mesolimbic reward pathway^34,35^. With its connectivity to reward processing regions^22,47^, anterior hippocampus may be particularly influenced by reward motivation. Anticipation of primary (e.g., food) or secondary (e.g., money) rewards activates mesolimbic regions, such as the substantia nigra, ventral tegmental area, and nucleus accumbens. These structures, in turn, project to anterior hippocampus^3,22,47,48^. As such, one putative mechanism for reward-enhancing effects on episodic memory is the tandem activation of mesolimbic structures and anterior hippocampus while anticipating rewards during encoding.

Based on dopaminergic effects of reward on memory and longitudinal specialization of the hippocampus, reward at encoding may exert specific influence on gist-based memory processes as both converge on anterior hippocampus. However, it should be noted that there is not definitive evidence to suggest that posterior HPC is not influenced by dopaminergic or noradrenergic connections to anterior HPC. It is possible that there are downstream effects from reward modulation at anterior hippocampus that affect posterior hippocampus as well.

### The current study

The preceding review of the literature has identified two factors that influence memory. First, according to the levels of processing account, encoding and subsequent recognition of information will benefit from deeper, semantic levels of processing (i.e., gist) compared to shallower, perceptual levels of processing (i.e., details). Second, motivation and arousal associated with reward anticipation boosts encoding and memory for task-relevant information. It is unclear, however, which level of processing is influenced by reward motivation. There are three potential scenarios regarding the interaction of levels of processing and reward. One possibility is that reward anticipation and levels of processing do not interact, and gist-level processing and reward motivation boost encoding in an additive manner. Another possibility is that reward, with its capacity to narrow attention, dampens the levels-of-processing effect and selectively boosts detail-level processing. This scenario would lead to equivalent memory outcomes for detail and gist-encoded information. Lastly, consistent with the proposal of functional specialization along the hippocampal long axis, it is possible that reward motivation amplifies the levels-of-processing effect. This scenario would lead to a selective enhancement of memory for gist-level representations.

**Figure 1:**
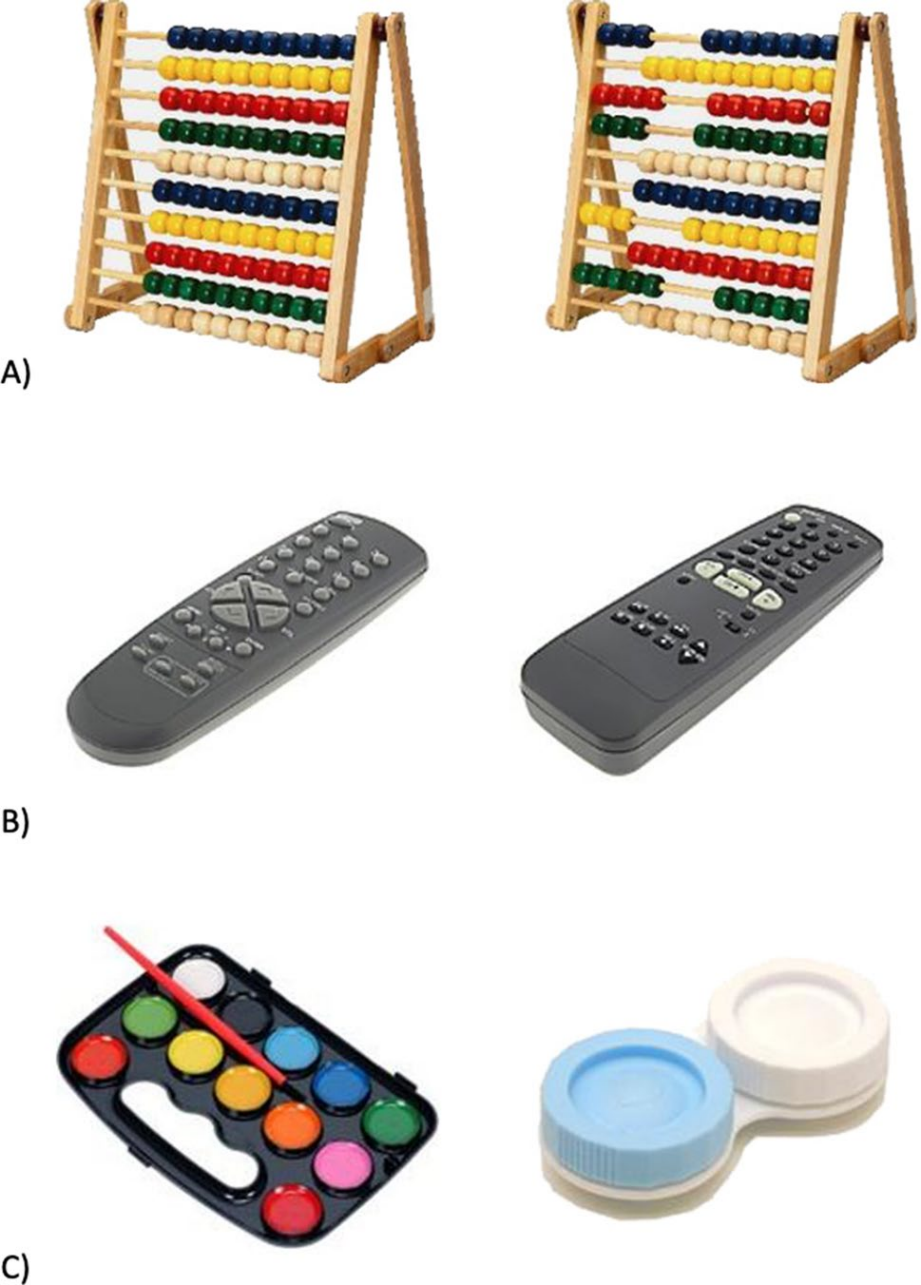
Examples of object simuli taken from Brady et al. database representing three distinct pair types. (**A**) “state” stimulus pair; (**B**) “exemplar” stimulus pair; (**C**) “novel" stimulus pair. Object images taken from Brady et al.^1^, available from https://bradylab.ucsd.edu/stimuli.html.

To adjudicate among these predictions, we gave participants an incidental encoding task in which they made judgments about pairs of objects (see “Method” section, Fig. 1). The first manipulation was that the judgment required either attention to semantic gist (i.e., Are these objects from the same category?) or attention to perceptual detail (i.e., Are these objects identical to one another?). The 144 object pairs were constructed so that “same” and “different” responses were equally likely overall within both the gist and detail conditions. The second manipulation was that reward anticipation was varied across trials using high-value and low-value cues. By crossing the two encoding manipulations (attention to gist-vs-detail; high vs. low reward anticipation) and assessing subsequent memory performance, we sought to test competing hypotheses about the effects of reward anticipation on gist and detail representations. Based on the levels-of-processing framework and reward-motivated memory, we expected that memory would benefit from both gist-level processing and high-reward at encoding. Moreover, based on and functional specialization of the hippocampus, we expected that reward would provide a specific boost to gist-level processing at encoding, amplifying the levels of processing effect in the high-reward condition.

## Results

For the encoding task, dependent variables were mean accuracy and median reaction time for correct responses. For the encoding judgments, accuracy was defined as the proportion of correct same/different responses. For the flanker trials, accuracy was defined as the proportion of correct left/right responses. Examination of these measures provided information about the difficulty and the attentional demands of the different encoding conditions.

In the recognition task, dependent variables were accuracy (hit rate minus false-alarm rate), hit rate, and median reaction time for correct responses. The hit rate was calculated as the total number of recognition hits divided by the total number of old trials. Hits were defined as “R”, “5”, or “4” responses to old objects, whereas false alarms were defined as “R”, “5”, or “4” responses new objects^2^. While accuracy provide information about overall recognition performance, hit rates were informative about differences related to the four encoding conditions. False alarm rate, which is only calculated with respect to new items, cannot be linked to a particular encoding judgment or reward level. Therefore, hit rate was used as the primary dependent measure for main analyses instead of accuracy. This approach has been used in prior studies where the key manipulations are introduced at encoding^3,22,25,26,49^.

Accuracy (encoding task), hit rate (recognition task) and reaction time (encoding and recognition tasks) were analyzed using separate 2 (reward: high, low) × 2 (encoding judgment: gist vs. detail) within-subjects analyses of variance (ANOVAs). For the flanker performance, flanker type (congruent, incongruent) was included as an additional within-subjects factor, resulting in a 2 × 2 × 2 within-subjects design. Significant interactions were probed using simple effects. Descriptive statistics of encoding and recognition performance are reported in Table 1.

**Table 1:** Descriptives from task performance.

	Overall		LR/gist		HR/gist		LR/detail		HR/detail	
**Encoding**										
Reaction time (ms)	514	(127)	509	(134)	508	(134)	521	(121)	517	(122)
Accuracy	0.79	(0.11)	0.82	(0.12)	0.83	(0.12)	0.77	(0.13)	0.77	(0.16)
**Recognition**										
Reaction time (ms)	1391	(501)	1427	(532)	1390	(467)	1369	(517)	1379	(498)
Hit rate	0.68	(0.14)	0.62	(0.17)	0.73	(0.13)	0.70	(0.09)	0.69	(0.13)
False alarm rate	0.14	(0.15)	–	–	–	–	–	–	–	–
**Signal detection**										
*d'*	0.19	(1.15)	–	–	–	–	–	–	–	–
*C*	0.13	(0.82)	–	–	–	–	–	–	–	–

*HR* high reward. *LR* low reward.

Values represent means, with standard deviations shown in parentheses. Mean reaction times were calculated from participants’ median reaction times for correct trials in each condition.

### Encoding task: judgment performance

#### Accuracy

Accuracy scores were submitted to a 2 (encoding judgment: gist, detail) × 2 (encoding reward: high, low) within-subjects ANOVA. Results revealed a main effect of encoding judgment, *F*(1,49) = 10.41, *p* < 0.01, *η*_*p*_^2^ = 0.18, such that accuracy was higher in the gist condition (*M* = 0.82, *SD* = 0.11) than in the detail condition (*M* = 0.77, *SD* = 0.14). There was no main effect of reward, *F*(1,49) = 0.39, *p* = 0.53, *η*_*p*_^2^ = 0.01, and no significant Encoding Judgment x Reward interaction, *F*(1,49) = 0.18, *p* = 0.67, *η*_*p*_^2^ < 0.01.

#### Reaction time

Median reaction time was submitted to a 2 (encoding judgment: gist, detail) × 2 (reward: high, low) within-subjects ANOVA. Results revealed no main effect of encoding judgment, *F*(1,49) = 1.78, *p* = 0.19, *η*_*p*_^2^ = 0.04, or reward, *F*(1,49) = 0.13, *p* = 0.72, *η*_*p*_^2^ < 0.01. Likewise, there was no significant Encoding Judgment x Reward interaction, *F*(1,49) = 0.01, *p* = 0.91, *η*_*p*_^2^ < 0.01.

In summary, gist-level judgments were more accurate than detail-level judgments, but there was no evidence for an effect of reward anticipation on accuracy or reaction times for the encoding judgments.

### Encoding task: flanker performance

#### Accuracy

Accuracy was submitted to a 2 (encoding judgment: gist, detail) × 2 (encoding reward: high, low) × 2 (flanker type: congruent, incongruent) within-subjects ANOVA. Results revealed no main effect of encoding judgment, *F*(1,49) = 1.46, *p* = 0.23, *η*_*p*_^2^ = 0.03, reward, *F*(1,49) = 0.87, *p* = 0.36, *η*_*p*_^2^ = 0.02, or flanker type, *F*(1,49) = 0.63, *p* = 0.43, *η*_*p*_^2^ = 0.01. Additionally, there were no significant higher-order interactions; encoding judgment × reward, *F*(1,49) = 0.01, *p* = 0.91, *η*_*p*_^2^ < 0.01; encoding judgment × flanker type, *F*(1,49) = 0.04, *p* = 0.84, *η*_*p*_^2^ < 0.01; reward × flanker type, *F*(1,49) = 1.78, *p* = 0.19, *η*_*p*_^2^ = 0.04; encoding judgment × reward × flanker type, *F*(1,49) = 0.16, *p* = 0.69, *η*_*p*_^2^ < 0.01.

#### Reaction time

Median reaction time was submitted to a 2 (encoding judgment: gist, detail) × 2 (reward: high, low) × 2 (flanker type: congruent, incongruent) within-subjects ANOVA. Results revealed only a main effect of flanker type, *F*(1,49) = 27.84, *p* < 0.01, *η*_*p*_^2^ = 0.36, indicating that reaction times were shorter on trials with congruent flankers (*M* = 446 ms, *SD* = 98 ms) compared with incongruent flankers (*M* = 501 ms, *SD* = 73 ms). There were no main effects of encoding judgment, *F*(1,49) = 0.91, *p* = 0.35, *η*_*p*_^2^ = 0.02, or reward, *F*(1,49) = 0.05, *p* = 0.82, *η*_*p*_^2^ < 0.01. Likewise, there were no significant higher-order interactions. encoding judgment × reward, *F*(1,49) = 0.51, *p* = 0.48, *η*_*p*_^2^ = 0.01; encoding judgment × flanker type, *F*(1,49) = 0.89, *p* = 0.35, *η*_*p*_^2^ = 0.02; reward × flanker type, *F*(1,49) = 1.57, *p* = 0.22, *η*_*p*_^2^ = 0.03; encoding judgment × reward × flanker type, *F*(1,49) = 0.93, *p* = 0.34, *η*_*p*_^2^ = 0.02.

Overall, the analysis of the flanker responses shows the expected congruency effect on reaction time. Critically however, neither accuracy nor reaction time on flanker trials was modulated by encoding judgment (category vs. object) or reward anticipation (high vs. low), contrary to what would have been expected if these manipulations had differed in their attentional demands.

### Recognition task

#### Accuracy

**Figure 2:**
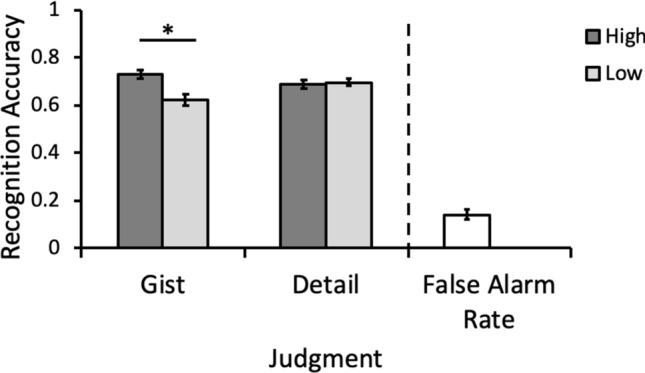
Recognition hit rate for old items according to judgment type and reward level at encoding and overall false alarm rate. For gist-encoded objects, the hit rate was higher for objects encoded during high vs. low reward anticipation. For detail-encoded objects, hit rate was not sensitive to the level of reward at encoding. *Significant at the level of p < 0.001.

The mean hit rate was 67.89% (*SD* = 0.14) and the mean false-alarm rate was 10.68% (*SD* = 0.07), corresponding to an overall recognition accuracy of 57.21% (*SD* = 0.13). Because the different encoding manipulations were not associated with separate false-alarm rates, only the hit rate was used to assess the effect of these manipulations on recognition memory. We submitted the hit rate to a 2 (encoding judgment: gist, detail) × 2 (reward: high, low) within-subjects ANOVA. Results revealed no main effect of encoding judgment, *F*(1,49) = 1.44, *p* = 0.24, *η*_*p*_^2^ = 0.03, but there was a significant main effect of reward, *F*(1,49) = 14.68, *p* < 0.01, *η*_*p*_^2^ = 0.23. The hit rate for objects encoded during anticipation of high reward (*M* = 0.71, *SD* = 0.16) was higher than the hit rate for objects encoded during anticipation of low reward (*M* = 0.66, *SD* = 0.15). This main effect of reward was qualified by a significant Encoding Judgment × Reward interaction, *F*(1,49) = 30.68, *p* < 0.01, *η*_*p*_^2^ = 0.39 (see Fig. 2). Simple effects revealed that, for objects from gist-based encoding judgments, hit rate was higher for objects encoded during anticipation of high reward (*M* = 0.73, *SD* = 0.13) versus low reward (*M* = 0.62, *SD* = 0.17). However, for objects from detail-based encoding judgments, hit rate was similar for objects encoded during anticipation of high reward (*M* = 0.69, *SD* = 0.09) versus low reward (*M* = 0.70, *SD* = 0.13). The same pattern of results was observed for hit rates of recollection-based responses (“R” rating) and for hit rates of familiarity-based responses (pooling across “4” and “5” ratings). Results from these analyses are reported in the Supplementary Information file (see Supplementary Table S1, Figs. S1, S2).

#### Reaction time

Median reaction time was submitted to a 2 (encoding judgment: gist, detail) × 2 (reward: high, low) within-subjects ANOVA. Results revealed no main effect of encoding judgment, *F*(1,49) = 2.17, *p* = 0.15, *η*_*p*_^2^ = 0.04, or reward, *F*(1,49) = 0.65, *p* = 0.43, *η*_*p*_^2^ = 0.01. There was also no significant Encoding Judgment x Reward interaction, *F*(1,49) = 0.81, *p* = 0.37, *η*_*p*_^2^ = 0.02.

In summary, at retrieval, participants’ response speed was not modulated by encoding judgment or reward. However, there was a reward-related boost in recognition hit rate for objects encoded with attention to gist but not for objects encoded with attention to detail.

## Discussion

The current study examined the influence of reward anticipation on the encoding of gist versus detail. While there is a well-documented benefit of reward anticipation for memory^2,21,22^, it has been unclear whether this benefit affects all levels of memory representations. Based on levels of processing, dopaminergic and noradrenergic effects of reward, and hippocampal specialization, we predicted that reward anticipation should selectively benefit encoding of gist information. To test this prediction, we asked participants to make “category” or “object” judgments at encoding, thereby drawing attention to gist or detail-level information, respectively. Both the judgment type (gist/detail) and the level of reward anticipation (high/low) were manipulated within subjects, across the same set of memoranda.

Results demonstrated that items encoded in the gist condition were better remembered than items encoded in the detail condition. Critically, only the gist condition showed an effect of reward anticipation, with high-reward gist items more likely to be recognized than low-reward gist items. By contrast, there was no significant difference in recognition hit rates for high-reward and low-reward items from the detail condition. These findings suggest that reward anticipation at encoding boosts gist-based encoding processes, but not detail-based encoding processes. The selective effect of reward anticipation on encoding of gist-level representations is consistent with the theory of long-axis specialization of the human hippocampus^34,35^, which localizes gist-level representations to the anterior hippocampus, a region with rich connectivity to the dopaminergic reward network^48^.

Several aspects of the theory of long-axis hippocampal specialization are consistent with our results. First, the theory suggests that anterior hippocampus is more engaged than posterior hippocampus during encoding^50^. This is relevant because both manipulations—level of reward and level of representation—were manipulated at encoding rather than at retrieval. Second, due to distinct connectivity of anterior and posterior hippocampus, the theory suggests that anterior hippocampus is disproportionately involved in dopaminergic processing and reward motivation^51^. Third, the anterior hippocampus has been implicated in the storage of recent rather than remote memory representations^43^, which is consistent with the short time interval between encoding and retrieval in our design. Last, and most relevant to our attention manipulation at encoding, the anterior hippocampus is implicated in gist-level memory representations^52^.

Why might focusing on coarser-grained gist rather than finer-grained detail during motivational states benefit memory? Our interpretation of the results is that reward anticipation sets a motivational context that enhances attention to task-relevant information, which is gist-level information during “category” judgments and detail-level information during “object” judgments. However, this bolstered attention has different effects on later recognition, consistent with the levels of processing framework^4,5^. This framework predicts that memory benefits from semantic versus perceptual encoding. Heightened attention to gist information at encoding—a semantic level of processing—facilitates subsequent recognition because it leverages existing category-level schemas (e.g., holistic processing, conceptual hook^53^). Heightened attention to detail—a perceptual level of processing—precludes this type of integration and gives rise to a more fragmented object perception at encoding^54^. Thus, reward motivation enhances attention indiscriminately, but selectively benefits encoding and recognition of gist-encoded objects.

Many studies have documented better memory for gist memory representations or faster forgetting of verbatim memory traces (e.g., fuzzy-trace theory^15^) and better memory for information associated with high rather than low reward^55^. However, to our knowledge, no studies have investigated the interaction of benefits from reward and gist. Therefore, the current study provides added insight that increased recognition from focusing on gist at encoding, but not detail, is sensitive to level of reward anticipation. One possibility is that recognition tests (which may rely more on gist-like familiarity) provide an advantage for gist-encoded objects. This is unlikely for two reasons. First, there was no main effect of encoding judgment. Rather, gist-encoded objects were better remembered only when they were associated with high reward. Second, this recognition boost was observed for both recollection and familiarity-based responses^56^. While it does not appear that gist-encoded items had a specific task advantage, it is still possible that the current task was not optimal for detecting reward effects in detail-encoded items. In future work, it would be interesting to adapt the paradigm and test whether this boost to high-reward, gist-encoded information extends to other episodic memory tasks, such as source memory and recall tests.

We did not replicate the typical levels of processing effect, as there was no main effect of encoding judgment on subsequent recognition hit rate. This may be due to two reasons. First, the encoding task was not blocked with respect to encoding judgment condition. It is possible that intermixing levels of processing trials at encoding diluted the overall levels of processing effect and a blocked design would have revealed the canonical effect^13^. Second, and related to the first point, considering levels of processing are a fluid construct, it is likely that some degree of semantic gist was extracted from detail encoding trials, and vice versa.

The degree to which our key finding would generalize to other paradigms unknown. We would speculate that the finding is limited to incidental encoding paradigms, because intentional encoding strategies (e.g., self-referential processing, visualization) could override the effects of gist-level and detail-level encoding prompts. Likewise, the effect may not generalize to single-item encoding conditions, in which perceptual load is lower, allowing attention to spread across gist and detail levels^57^. In this case, we may not expect to see an interaction of reward anticipation with encoding judgment. It is also unclear how a longer retention interval would affect the pattern observed in the current study. Reward effects on episodic memory have been shown to strengthen with time for consolidation in both incidental^58^ and intentional^25^ memory paradigms. Detail-encoded items are more purely episodic in nature than gist-encoded items which can benefit from existing semantic category schemas^53^. Thus, it is possible that reward effects on detail-encoded items may appear later, after time allowed for consolidation.

Even without time for consolidation, it may seem surprising that reward anticipation offered no memory advantage to items from the detail-level encoding condition. We believe that this result provides insight into the nature of reward-driven benefits to incidental memory documented in other studies. Gist-level processing may be the default level of processing for visual objects on incidental encoding tasks. In our study, this hypothesis was supported by participants’ higher accuracy on gist-level judgments during the encoding task. Therefore, if participants typically adopt a gist level of analysis during incidental encoding, reward anticipation effects shown in prior work^2,3,26^ have demonstrated the same finding as the current study. That is, that high reward anticipation while processing visual objects at a categorical, gist-based level of analysis leads to better recognition. We argue that it is unlikely that participants in these studies processed objects in a detail-based manner without specific instruction to do so. For example, we automatically interpret the meaning of a full sentence rather than attend to constituent words. As such, our findings may shed light on the automaticity of gist-level encoding processes relative to detail-level encoding processes during incidental encoding tasks.

Another interesting finding was that familiarity and recollection demonstrated the same pattern of a gist-specific boost from reward at encoding. While not related to our main hypotheses, these results add some insight to the literatures on both levels-of-processing effects and reward effects on the dual processes of recognition. According to the dual process model, recognition is thought to comprise two processes, recollection and familiarity^56^. Recollection, a threshold process, is the high-fidelity, vivid recall of old information with specific detail while familiarity, a signal detection process, is a vague sense of knowing information is old without specific episodic detail^59^.

The memory advantage for gist over detail is a widespread and robust finding, but the question of the underlying mechanisms is not fully resolved. Some studies suggest that gist-level processing predicts better recollection and familiarity^13,60,61^, while others have shown that gist-level processing only boosts recollection^62,63^. It has also been suggested that gist-level and detail-level processing in tandem will lead to better recollective recognition^64^. We did not replicate the levels-of-processing effect on recognition which may indicate that both gist and detail levels of analysis contribute to recognition processes. It is possible that interleaving gist and detail level encoding judgements, rather than using a blocked design, diluted the levels of processing effect^13^. Thus, it would be interesting to examine differences in recollection and familiarity using a blocked version of the current encoding paradigm.

Few previous studies have directly addressed which aspects of recognition are boosted by motivation and arousal associated with reward anticipation. One study indicated that anticipation of monetary reward during an incidental encoding task selectively boosted recollection, but not familiarity, for high-reward visual objects^2^. Another study manipulated novelty anticipation, a motivational variable that recruits the reward system^65,66^, during intentional encoding of landscapes. Anticipation of novel landscapes during encoding provided a specific benefit to recollection. While affective valence is not equivalent to reward motivation, it does have arousing properties. In a study investigating the influence of valence and arousal on recollection and familiarity, results indicated that recollection benefitted specifically from low-arousal, positively-valenced stimuli whereas familiarity was not sensitive to valence or arousal^67^. We found a main effect of reward in the opposite direction, such that high reward selectively boosted familiarity (see Supplementary Information), which was driven by the gist encoding condition. Thus, our findings highlight the possibility that reward benefits the recognition process most consistent with the encoding process being enhanced (i.e., familiarity is more consistent with gist processing while recollection is more consistent with detail processing).

Several limitations should be noted for the current study. First, it did not include a baseline condition—i.e., a reward-free encoding condition that did not bias attention to either gist or detail. Instead, the low reward anticipation condition served as a within-subjects baseline. Relatedly, measures of motivation were purely based on subjective self-report in a post-task interview and so it is unclear how effective our motivation manipulation truly was. Whereas post-task interviews indicated that participants felt motivated by the virtual points, the behavioural data from the encoding phase showed no effect of reward on the speed or accuracy of responding. Another limitation related to the reward manipulation is the potential for lingering effects of reward on encoding for temporally-proximal trials^26^, which we did not investigate as it was peripheral to the central aims of the study and the task included feedback on only ~ 30% of trials. However, it is possible that trials following feedback screens with larger increases to the cumulative score were encoded differently than trials following feedback screens with smaller increases^68^. We also note the limited ability of our dichotomous encoding judgment manipulation (gist vs detail) to capture the full spectrum of levels of processing which is better conceptualized as a continuous variable. Future studies could use a more nuanced manipulation, orienting attention to multiple levels of perceptual detail and semantic gist (e.g., categorizing “same” items according to colour, position, function, and category). Additionally, we acknowledge that it is difficult to draw a firm line between perceptual and conceptual processing, as details may provide higher-level gist information (e.g., the colour of food indicating it is spoiled^69^), and categorical information can be leveraged to facilitate detail-oriented memory representations^53^. Thus, a more sensitive and intersectional measure of gist and detail processing could bypass these concerns. Lastly, the study used behavioural measures, rendering inferences about underlying neurobiological mechanisms somewhat speculative. Future studies could use a multimethod approach to directly measure motivation (i.e., pupillometry to measure arousal during reward anticipation^70^) and neural substrates associated with reward motivation, encoding and recognition of different levels of representation (e.g., fMRI to investigate the long-axis specialization of the hippocampus^71^).

In summary, the current results suggest that reward anticipation enhances memory for gist but does not boost memory detail. In addition to constraining theories of motivation-cognition interaction, these findings have implications for the utility of extrinsic rewards in educational contexts. Specifically, such incentives may be helpful in boosting memory for gist-based information (e.g., meanings and narratives), but may do little to enhance memory for perceptual details.

## Method

### Participants

Participants included 50 younger adults (17–35 years old; 33 female) recruited from the community via physical flyers and advertisements hosted on an online community advertisement platform (kijiji.com). The sample size was determined based on results of a power analysis run using G-Power software^72^ to achieve a power of at least 0.90 to detect a medium (*f* = 0.25) within-subjects main effect or interaction, assuming an alpha error probability of 0.05 and a correlation among levels of the within-subjects factors of 0.50 or higher. Inclusion criteria for the study included normal (or corrected-to-normal) vision and hearing and no presence of medical conditions that might affect cognitive ability such as neurological, psychiatric and/or cardiovascular disorders. A total of 52 participants were tested, but two were excluded due to technical issues during the testing session, leaving 50 participants included in the final analysis. Each participant received $15 CAD for participating. At the outset of the experimental session, informed consent was obtained from all participants, and from legally authorized persons of participants less than 18 years of age. All participants were debriefed at the end of the session. The study procedure was approved by the Ryerson University Research Ethics Board and the research was performed in accordance with the relevant guidelines and regulations from the board.

### Materials

Experimental tasks were programmed in E-Prime 2.0 software (Psychology Software Tools, Pittsburgh, PA) and were presented on a 17-inch computer display. During the tasks, participants made responses on a keyboard, using the spacebar to move through instruction screens and the arrow keys for the encoding task. For the recognition task, participants could respond with “1”, “2”, “3”, “4”, “5” or “R” on each trial. Text was black and set to size 32, Arial font. For the encoding task, trial stimuli were 144 object pairs drawn from Brady et al.’s^1^ object stimuli database (available from https://bradylab.ucsd.edu/stimuli.html). Using Brady et al.’s^1^ terminology, there were three types of object pairs, with 48 pairs of each type: (1) exemplar, with two objects from the same category (e.g., two nonidentical cabinets); (2) state, with two images of the same object in different configuration (e.g., a cabinet in opened vs. closed states); and (3) novel, with two unique objects (e.g., a cabinet and a sweater). See Fig. 1 for examples of each pair type. The inclusion of novel, exemplar and state pairs allowed for an equal distribution of “same” and “different” responses across the gist (same category?) and detail (same object?) judgment trials. For gist judgments, exemplar and state pairs required “same” responses, whereas novel pairs required “different” responses. For detail judgments, state pairs required “same” responses, whereas novel and exemplar pairs required “different” responses. In addition, there were 12 practice pairs which included 4 pairs each of exemplar, state, and novel objects.

For the recognition task, stimuli include 96 objects pseudo-randomly selected from the encoding task, as well as 48 new objects which were unrelated to the old study pairs. The 96 old objects included 32 exemplar, 32 state, and 32 novel objects from the encoding task, with 48 objects originating from gist judgment trials and 48 originating from detail judgment trials.

### Procedure

#### Encoding task

**Figure 3:**
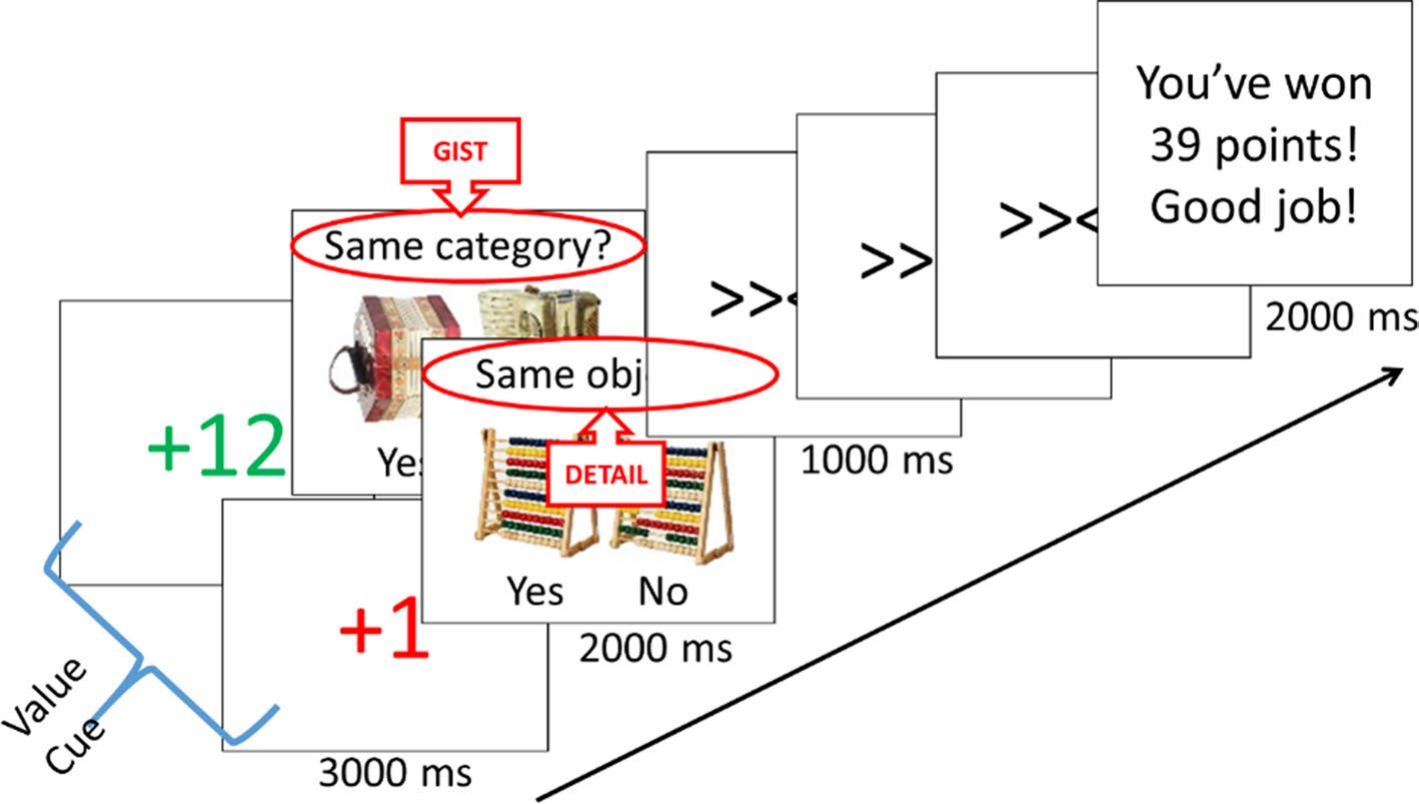
Schematic of trial sequence for the encoding task design. Participations saw high or low value cues before making a gist-level or detail-level judgment. Roughly 30% of trials included a feedback screen after flanker screens. Object images taken from Brady et al.^1^, available from https://bradylab.ucsd.edu/stimuli.html.

The incidental encoding task 12 practice trials followed by 144 experimental trials. The first and second half of the experimental trials were separated by a mandatory break (at least 30 s long). Each trial began with a value cue, indicating that correct judgment performance on the current trial would add a high point value (+ 10, 11, or 12) or a low point value (+ 1, 2, or 3) to the total score. The value cue lasted for 3000 ms and was followed by a 500 ms fixation cross, after which the judgment screen appeared. The judgment screen presented two objects side-by-side, accompanied by the question: “Same object?” or “Same category?” Participants had 2000 ms to view the object pair and respond with the left or right arrow keys corresponding to the words “same” or “different” presented on the left and right lower portion of the screen. After the encoding screen, there was another 500 ms fixation, followed by three screens of a flanker task, during which participants had 1000 ms to indicate the direction of the central arrow using the left or right arrow key. The flanker trials were used to assess lingering effects of reward on attention and as distractors to prevent any further elaborative processing of items from the preceding judgment screen. Flanker screens were followed by an inter-trial interval (blank screen) of 2000 ms or a feedback screen indicating the current cumulative point score. Feedback screens were programmed to occur every three trials on average such that each participant saw 48 inter-trial feedback screens across the entire task. Points were awarded based only on correct judgment performance, regardless of flanker performance. All participants saw a self-paced final feedback screen at the end of the task to view their overall point score. See Fig. 3 for a schematic of the encoding task.

#### Inter-task interval

In order to obscure the connection between the encoding and recognition tasks, participants completed a series of unrelated paper-and-pencil tasks during a 20-min interval.

#### Recognition task

**Figure 4:**
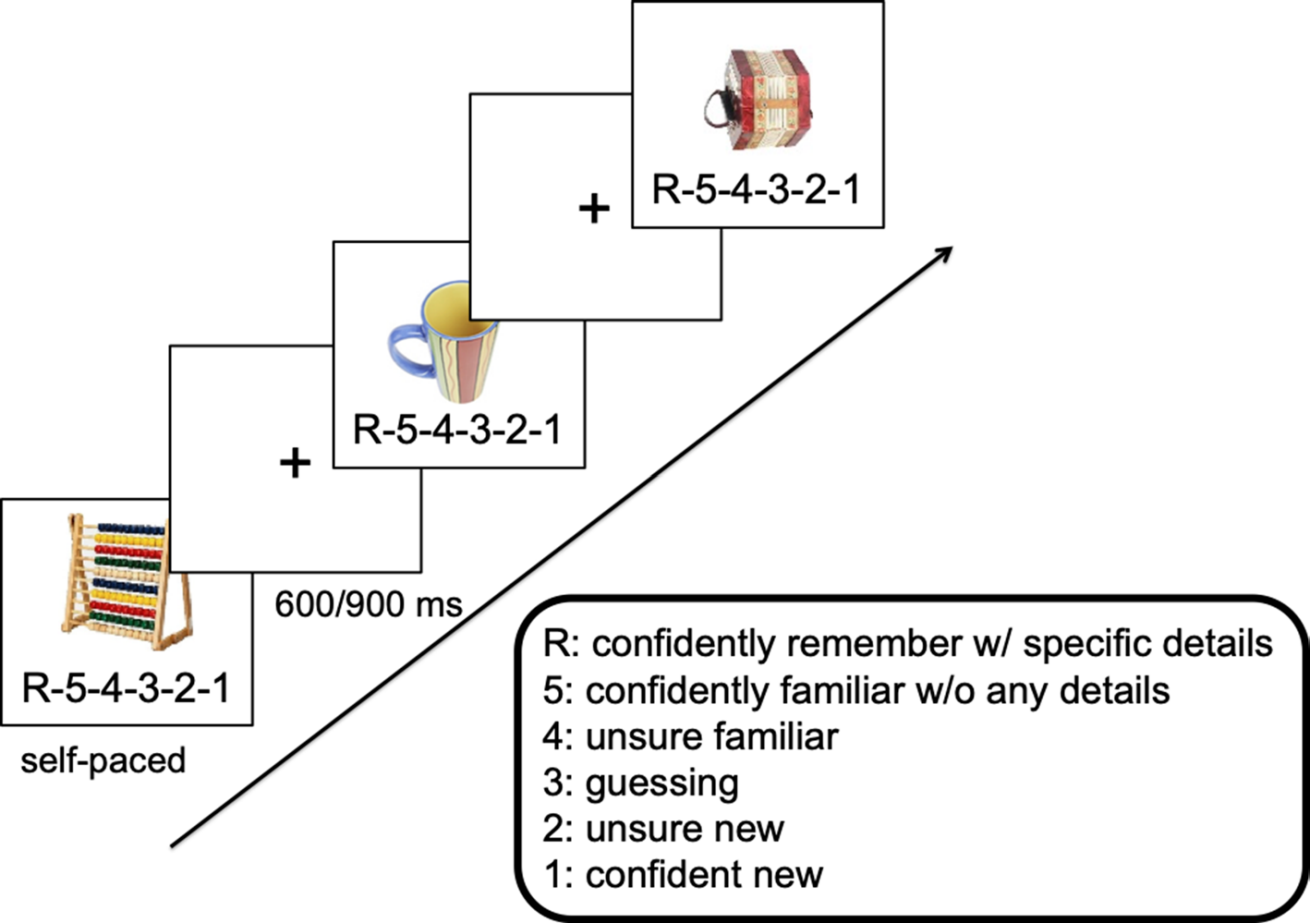
Schematic of retrieval task. Participants saw old or new object images and responded according to the scale pictured below the image. Object images taken from Brady et al.^1^, available from https://bradylab.ucsd.edu/stimuli.html.

The recognition task consisted of 144 trials plus 12 practice trials. The task began with instructions, followed by practice trials, followed by the experimental trials. Each trial consisted of an old or new object presented centrally on the screen until the participant made a response. A scale adapted from Gruber et al.^2^ was displayed underneath the object, and the participant made a response according to the scale. Pressing “R” indicated that the participant confidently remembered the object with vivid detail, “5” indicated that the participant confidently remembered the object without vivid detail, “4” indicated that the participant as unsure but felt the object was familiar, “3” indicated a guess, “2” indicated that the participant was unsure but felt it was a new object, and “1” indicated that the participant confidently identified the object as new. After a participant made a response, the screen advance to the next trial until all trials were complete. No feedback was provided during this task. See Fig. 4 for a schematic of the recognition task.

### Conference presentation

Findings from this study were presented at the 59th Annual Meeting of the Psychonomic Society in New Orleans, Louisiana (November, 2018).

## Supplementary information

Supplementary Information.
