# Supplementary material for: Reward anticipation selectively boosts encoding of gist for visual objects
Source: Sci Rep. 2020 Nov 19;10:20196. doi: 10.1038/s41598-020-77369-4 (PMC7677401; doi:10.1038/s41598-020-77369-4)
Supplement: Supplementary file 1 — Supplementary Information. [file 41598_2020_77369_MOESM1_ESM.docx]

Supplementary Information

**Reward Anticipation Selectively Boosts Encoding of Gist for Visual Objects**

Liyana T. Swirsky

Ryerson University

Ryan M. Marinacci

Ryerson University

Julia Spaniol

Ryerson University

**Recognition memory analysis: Recollection (“R”) and familiarity (“5” and “4”)**

We estimated recognition based on the approach used by Gruber et al. (2016) who developed the scale used in the retrieval task. See Supplementary Table 1 for all results related to this analysis. Because the same pattern of results for the Reward x Encoding Judgment interaction was found for recollection and familiarity and to maximize the number of observations, we combined correct “R”, “5” and “4” responses for recognition memory in analyses reported in the main text.

| *Supplementary Table S1*. Dual process analysis | | |
| --- | --- | --- |
| Recollection |  |  |
| Hit rate | 0.34 | (0.28) |
| FA rate | 0.04 | (0.04) |
| Accuracy | 0.30 | (0.24) |
| Familiarity |  |  |
| Hit rate | 0.35 | (0.27) |
| FA rate | 0.10 | (0.10) |
| Accuracy | 0.25 | (0.25) |
| Corrected Hit rate | 0.23 | (0.27) |
| Corrected FA rate | 0.10 | (0.10) |
| Corrected accuracy | 0.13 | (0.25) |

Recollection hit rate was computed as the proportion of old item trials correctly recognized with an “R” response. Recollection false alarm rate was computed as the proportion of new item trials associated with an “R” response. Overall recollection accuracy was computed as recollection hit rate minus recollection false alarm rate. Familiarity hit rate was computed as the proportion of old trials correctly recognized with a “5” or “4” response. This value was then corrected by multiplying by (1 – recollection hit rate) to measure familiarity in the absence of recollection (Yonelinas and Jacoby, 1995). Likewise, familiarity false alarm rate was computed as the proportion of new trials associated with a “5” or “4” response, and this was corrected for recollection by multiplying by (1 – recollection false alarm rate).

To assess the effect of the encoding manipulations on recollection and familiarity, both hit rates were submitted to separate 2 (encoding judgment: gist, detail) x 2 (reward: high, low) within-subjects ANOVAs.

For recollection, results revealed no main effect of encoding judgment, *F*(1,49) = .28, *p* = .60, *η_p_^2^ <* .01, and no main effect of reward, *F*(1,49) = 2.68, *p* = .11, *η_p_^2^ =* .05. The Encoding Judgment x Reward interaction was significant, *F*(1,49) = 4.42, *p* = .04, *η_p_^2^ =* .08 (see Supplementary Figure 1a). Simple effects revealed that, for objects from gist-based encoding judgments, recollection hit rate was higher for objects encoded during anticipation of high reward (*M* = .37, *SD* = .30) versus low reward (*M* = .31, *SD* = .27). However, for objects from detail-based encoding judgments, recollection hit rate was similar for objects encoded during anticipation of high reward (*M* = .35, *SD* = .28) versus low reward (*M* = .36, *SD* = .28).

For familiarity, results revealed no main effect of encoding judgment, *F*(1,49) = 0.06, *p* = .80, *η_p_^2^ <* .01, but there was a significant main effect of reward, *F*(1,49) = 4.37, *p* = .04, *η_p_^2^ =* .08. The familiarity hit rate for objects encoded during anticipation of high reward (*M* = .36, *SD* = .25) was higher than the familiarity hit rate for objects encoded during anticipation of low reward (*M* = .33, *SD* = .25). This main effect of reward was qualified by a significant Encoding Judgment x Reward interaction, *F*(1,49) = 4.14, *p* = .05, *η_p_^2^ =* .08 (see supplementary Figure 1b). Simple effects revealed that, for objects from gist-based encoding judgments, familiarity hit rate was higher for objects encoded during anticipation of high reward (*M* = .38, *SD* = .29) versus low reward (*M* = .31, *SD* = .24). However, for objects from detail-based encoding judgments, familiarity hit rate was similar for objects encoded during anticipation of high reward (*M* = .34, *SD* = .28) versus low reward (*M* = .34, *SD* = .27).

A)


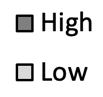


*

B)

*

*Supplementary Figure S1.* A) Recollection hit rate for old items according to encoding conditions of judgment type (gist, detail) and reward level (high, low) and recollection false alarm rate. B) Familiarity hit rate according to encoding conditions and familiarity false alarm rate. *significant at the level of p < .05

**R vs. 4/5 analysis: Encoding Judgment x Reward x Recognition Type**

Recognition hit rate was submitted to a 2 (encoding judgment: gist, detail) x 2 (reward: high, low) x 2 (recognition type: familiarity, recollection) within-subjects ANOVA. Results revealed no main effect of encoding judgment, *F*(1,49) = .13, *p* = .72, *η_p_^2^ <* .01, and no main effect of recognition type, *F*(1,49) = .01, *p* = .94, *η_p_^2^ <* .01. There was, however, a significant main effect of reward, *F*(1,49) = 5.57, *p* = .02, *η_p_^2^ =* .10. Regardless of encoding judgment or recognition type, the hit rate for objects encoded during anticipation of high reward (*M* = .36, *SD* = .29) was higher than the hit rate for objects encoded during anticipation of low reward (*M* = .33, *SD* = .26). This main effect of reward was qualified by a significant Encoding Judgment x Reward interaction, *F*(1,49) = 9.31, *p* < .01, *η_p_^2^ =* .16 (see supplementary Figure 2). Simple effects revealed that, for objects from gist-based encoding judgments, hit rate was higher for objects encoded during anticipation of high reward (*M* = .38, *SD* = .29) versus low reward (*M* = .31, *SD* = .25). However, for objects from detail-based encoding judgments, hit rate was similar for objects encoded during anticipation of high reward (*M* = .35, *SD* = .28) versus low reward (*M* = .35, *SD* = .27). No other interactions were significant; Encoding Judgment x Recognition Type, *F*(1,49) = .32, *p* = .58, *η_p_^2^ =* .01; Reward x Recognition Type, *F*(1,49) = .11, *p* = .75, *η_p_^2^ <* .01; Encoding Judgment x Reward x Recognition Type, *F*(1,49) = .03, *p* = .87, *η_p_^2^ <* .01.


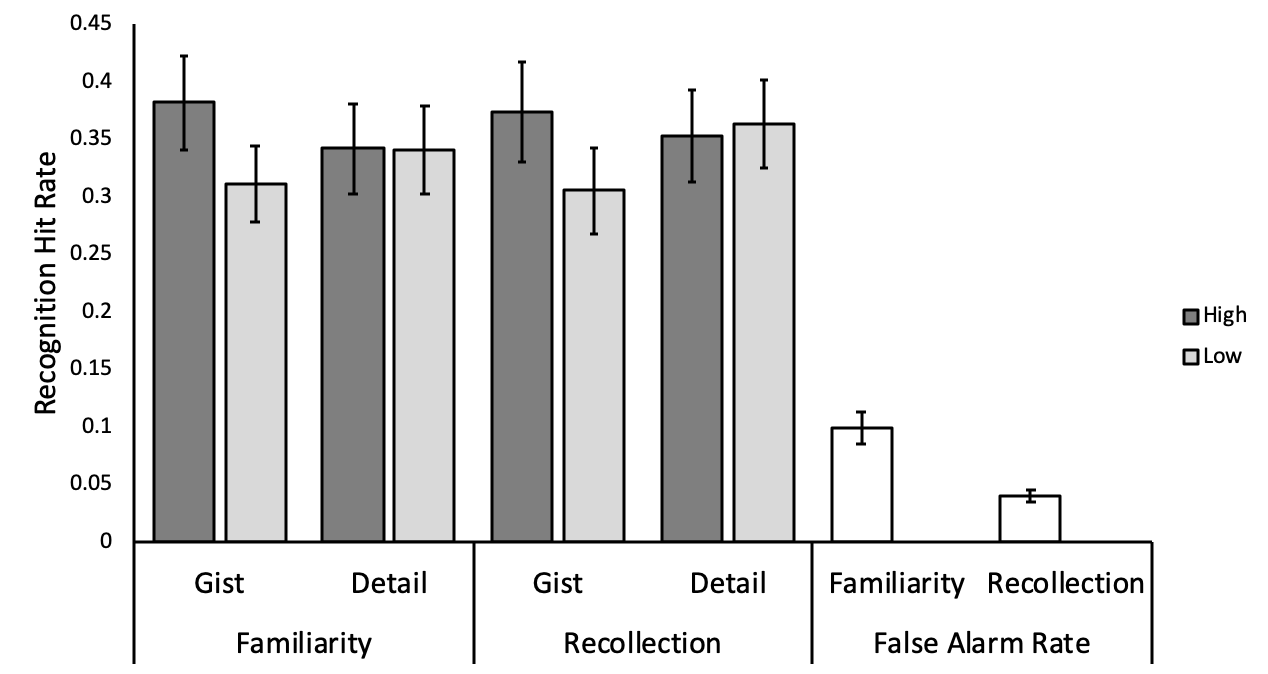


*

*

*Supplementary Figure S2.* Hit rate for old items according to encoding conditions of judgment type (gist, detail) and reward level (high, low) as well as recognition type (familiarity, recollection). False alarm rates are also displayed according to recognition type. *significant at the level of .05

**Item Type analysis: Encoding judgment x Reward x Item type x Reward**

We submitted the hit rate to a 2 (encoding judgment: gist, detail) x 2 (reward: high, low) x 3 (item type: exemplar, state, novel) within-subjects ANOVA (see supplementary Figure 3). Results revealed a significant main effect of item type, *F*(2,98) = 223.79, *p* < .01, *η_p_^2^ =* .82, such that state items (*M* = .84, *SD* = .17) were recognized significantly more than exemplar items (*M* = .73, *SD* = .18), which were remembered significantly more than novel items (*M* = .48, *SD* = .23). There was also a significant main effect of reward, *F*(1,49) = 12.84, *p* < .01, *η_p_^2^ =* .21, such that the hit rate for objects encoded during anticipation of high reward (*M* = .71, *SD* = .16) was higher than the hit rate for objects encoded during anticipation of low reward (*M* = .66, *SD* = .15). There was no main effect of encoding judgment, *F*(1,49) = .48, *p* = .49, *η_p_^2^ =* .01.

The main effect of reward was qualified by a significant Encoding Judgment x Reward interaction, *F*(1,49) = 20.74, *p* < .01, *η_p_^2^ =* .30 (see Figure 4, main text). Simple effects revealed that, for objects from gist-based encoding judgments, hit rate was higher for objects encoded during anticipation of high reward (*M* = .73, *SD* = .13) versus low reward (*M* = .62, *SD* = .17). However, for objects from detail-based encoding judgments, hit rate was similar for objects encoded during anticipation of high reward (*M* = .69, *SD* = .09) versus low reward (*M* = .70, *SD* = .13). There were no other significant interactions. Encoding Judgment x Item Type, *F*(2,48) = 1.14, *p* = .33, *η_p_^2^ =* .05; Reward x Item Type, *F*(2,48) = .54, *p* = .59, *η_p_^2^ =* .02; Encoding Judgment x Reward x Item Type, *F*(2,48) = .15, *p* = .86, *η_p_^2^ =* .01.

*

*

*

*Supplementary Figure S3.* Hit rate for old items according to encoding conditions of judgment type (gist, detail), reward level (high, low) and item type (state, novel, exemplar). State items were recognized most accurately, followed by exemplar items, followed by novel items. Regardless of item type, the reward-related boost in recognition is limited to items encoded with attention to gist*significant at the level of .001

**Feedback effects on item recognition**

Hit rate was submitted to a 2 (reward: low, high) x 3 (feedback: none, current, previous) within-subjects ANOVA. Results revealed no main effect of reward level on item hit rate, *F*(1,49) < .01, *p* = .96, *η_p_^2^ <* .01, and no main effect of encoding feedback on item hit rate, *F*(2,48) = .32, *p* = .73, *η_p_^2^ =* .01. Likewise there was no significant Reward x Feedback interaction, *F*(2,48) = .54, *p* = .58, *η_p_^2^ =* .02. Hit rates for items from the high and low reward encoding conditions were similar regardless of whether items were encoded on trials with no proximity to feedback (low reward: *M* = .68, *SD* = .17; high reward: *M* = .69, *SD* = .17), trials preceding feedback (low reward: *M* = .68, *SD* = .16; high reward: *M* = .67, *SD* = .17), and trials following feedback (low reward: *M* = .68, *SD* = .17; high reward: *M* = .69, *SD* = .15).
